# A single cooperative experience increases corticolimbic synaptic density and prosocial behavior

**DOI:** 10.64898/2026.09.07.749944

**Published:** 2026-09-14

**Authors:** Henry W. Kietzman, R. Allie Cauchon, Amelia Johnson, David R. Backer Peral, Robin Bonomi, Yiyun Huang, Hayde Sanchez, Shreya Saxena, S. William Li, Jane R. Taylor

## Abstract

Cooperation is typically studied as an output of the social brain, as opposed to an experience that shapes it. Here, we developed a task in rats that permits interaction while requiring joint action for reward. Cooperation depended on dyad familiarity and visual access. Anterior cingulate cortex (ACC) to basolateral amygdala (BLA) projections were activated during cooperation with a familiar partner, whereas ACC to anterior insula (AI) projections were simultaneously suppressed regardless of familiarity. A single 90 min cooperative experience increased presynaptic marker SV2A – detected by positron emission tomography (PET) – in the amygdala and insula one day later. Concurrently, rodents demonstrated heightened attention toward a distressed conspecific. Cooperation is thus an experience that recruits dissociable corticolimbic pathways and changes brain and behavior beyond the encounter itself.

## Full Text Availability

The license terms selected by the author(s) for this preprint version do not permit archiving in PMC. The full text is available from the preprint server.

